# Structure and function of the blood–brain barrier in perioperative neurocognitive disorders

**DOI:** 10.3389/fnins.2025.1690354

**Published:** 2026-01-02

**Authors:** Yipeng Su, Yiting Chen, Bingqi Zheng, Yiting Huang, Zhongxiao Liao, Xiaochun Zheng, Fei Gao

**Affiliations:** 1 Shengli Clinical Medical College, Fujian Medical University, Fuzhou, China; 2 School of Basic Medicine, Fujian Medical University, Fuzhou, China; 3 Fujian Provincial Hospital, Fuzhou University Affiliated Provincial Hospital, Fuzhou, China

**Keywords:** perioperative neurocognitive disorders, blood–brain barrier, endothelial cells, neuroinflammation, pathogenesis

## Abstract

**Background:**

Perioperative neurocognitive disorders (PNDs) are the most common neurological complications in elderly patients undergoing surgery. Patients with PNDs have significantly greater incidences of postoperative disability and mortality. Currently, there are no specific treatments for PNDs.

**Methods:**

This review integrates the latest evidence examining the role of structural and functional changes in the blood–brain barrier (BBB) in the pathological mechanisms of PNDs, with the aim of identifying innovative preventive strategies and promising therapeutic targets for PNDs.

**Results:**

Researchers have proposed various hypotheses to shed light on the pathogenesis of PNDs, including neuroinflammation, neurotransmitter or receptor abnormalities, beta-amyloid (Aβ) deposition and tau protein phosphorylation, oxidative stress, iron homeostasis imbalance, circadian rhythm disruption, and changes in the gut microbiota. Damage to the BBB plays a critical role in the pathogenesis of PNDs.

**Conclusion:**

This article summarizes the role of BBB structural and functional changes in the pathogenesis of PNDs reported in recent studies, with the goal of providing new ideas for preventing and treating PNDs.

## 1 Introduction

Perioperative neurocognitive disorders (PNDs) refer to neuropsychological changes occurring during the perioperative period, including preoperative cognitive impairment, postoperative delirium, delayed neurocognitive recovery, and postoperative cognitive dysfunction (POCD) (Evered et al., 2018). PNDs are commonly observed in elderly individuals, with 25.8% of patients experiencing some degree of cognitive impairment within 1 week postoperatively, and 9.9% of elderly patients having cognitive impairment lasting longer than 3 months (Moller et al., 1998; Evered et al., 2022). PNDs can prolong hospital stays and are closely associated with increased risks of dementia and long-term mortality, imposing a significant burden on patients’ families and society (Evered et al., 2022). As no established preventive or treatment methods for PNDs are currently available, effective therapeutic strategies targeting their pathophysiological mechanisms must be urgently explored.

**FIGURE 1:**
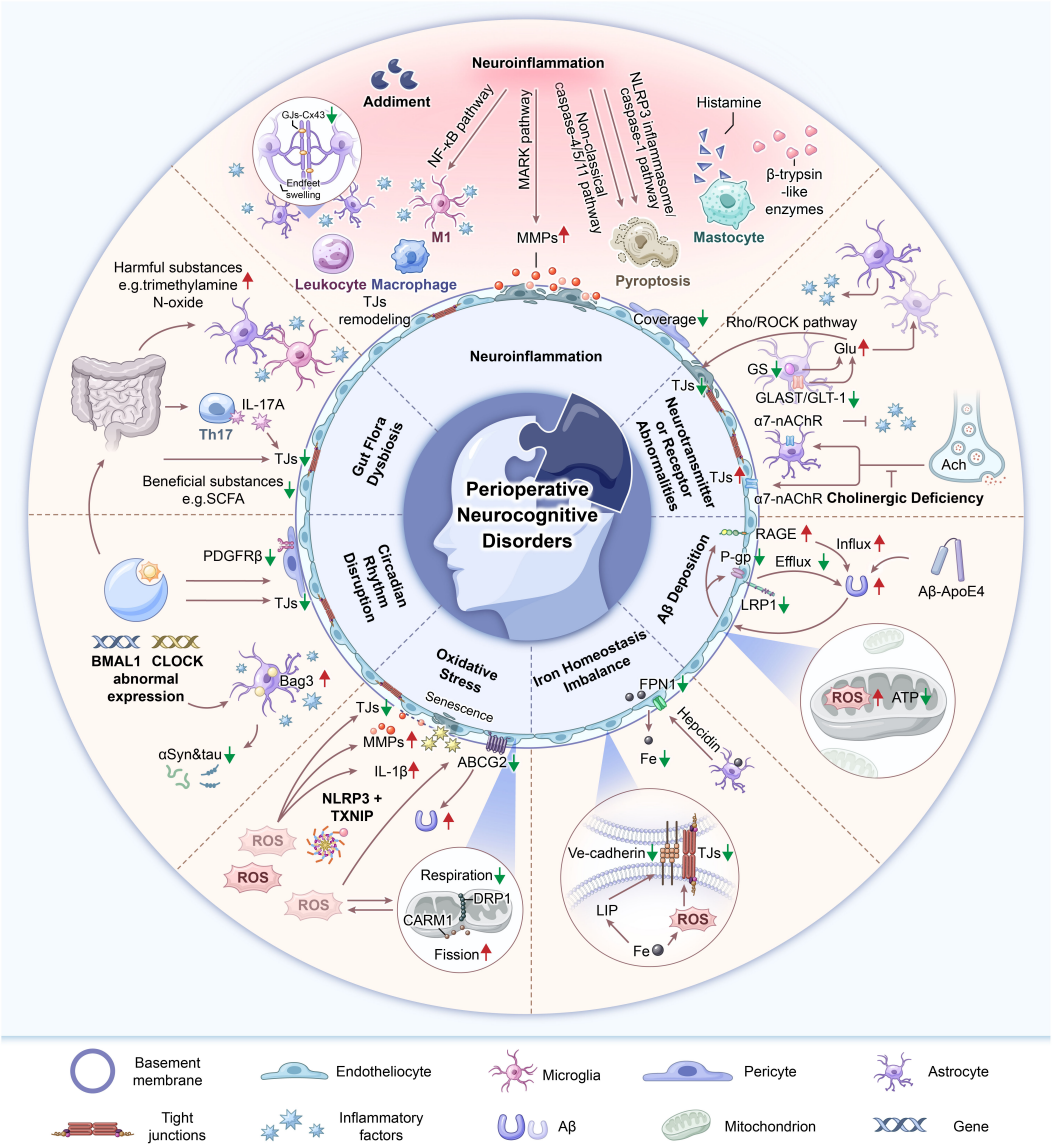
Diagram for the pathogenesis of perioperative neurocognitive disorders (PNDs), including neuroinflammation, neurotransmitter or receptor abnormalities, amyloid-β (Aβ) deposition, oxidative stress, iron homeostasis imbalance, circadian rhythm disruption, and gut flora dysbiosis.

Disruption and dysfunction of the blood–brain barrier (BBB) are considered critical to the onset and progression of PNDs (Ni et al., 2018; Yuan et al., 2023). The BBB comprises endothelial cells, basement membranes, pericytes, tight junctions (TJs), and astrocyte peduncles and forms a selective barrier that maintains central nervous system homeostasis and prevents peripheral toxins and pathogens from entering (Kadry et al., 2020). Pathological mechanisms linked to PNDs, such as neuroinflammation, neurotransmitter or receptor abnormalities, β-amyloid (Aβ) deposition, tau protein phosphorylation, oxidative stress, iron homeostasis imbalance, circadian rhythm disruption, and changes in the gut microbiota, are believed to be associated with BBB damage. Specifically, during the development of PNDs, neuroinflammation is connected with a decrease in BBB pericyte coverage (Yuan et al., 2023), circadian rhythm disruption can influence astrocyte activation (Hou et al., 2019), gut microbiota dysbiosis can alter the expression of TJs (Wen et al., 2020), and Aβ can also damage brain microvascular endothelial cells (Zhao et al., 2025). Elevated serum levels of the inflammatory markers high-mobility group box 1 (HMGB1) and interleukin (IL)-6 in patients after major gastrointestinal surgery are associated with damage to the BBB and the development of PNDs (Lin et al., 2014). This evidence suggests that BBB damage and dysfunction are critical components of the multiple pathological mechanisms underlying PNDs. Reviews on PNDs over the past 2 years have predominantly focused on individual mechanisms, such as neuroinflammation and mitochondrial dysfunction (Liu et al., 2023, 2025), or centered on specific cell types, like microglia and neutrophils (Liu and Zhang, 2025; Cheng et al., 2025). However, the BBB is generally addressed only as a critical component of neuroinflammation, overlooking its multifaceted roles in PNDs. Therefore, this paper adopts a BBB-centric approach to synthesizing evidence that links to multiple PND mechanisms and BBB impairment. We propose that BBB impairment acts as a central hub where these pathological pathways converge. This framework not only provides a more integrated understanding of PND pathogenesis but also highlights novel therapeutic targets and preventive strategies aimed at preserving BBB function (Figure 1).

## 2 Neuroinflammation and the BBB

Perioperative systemic inflammation disrupts the BBB, allowing peripheral inflammatory signals to enter the central nervous system. This activates and amplifies inflammatory responses within the multicellular networks of the neurovascular unit (NVU), ultimately damaging synapses and neural networks, manifesting as PNDs. BBB dysfunction facilitates the entry of peripheral inflammatory factors into the central nervous system while simultaneously promoting the maintenance and amplification of central inflammation.

Surgery and anesthesia can trigger systemic inflammation, disrupting the BBB. Peripheral inflammatory factors cross this barrier into the central nervous system, activating signaling pathways such as the nuclear factor-kappa B (NF-κB) pathway, inducing microglial polarization toward the pro-inflammatory M1 phenotype. Inflammation also activates the NOD-like receptor pyrin domain-containing 3 (NLRP3) inflammasome/caspase-1 axis and non-canonical caspase-4/5/11 pathways, contributing to the development of PNDs through inflammatory programmed cell death (pyroptosis) (Zhang and Yin, 2023; Cheng et al., 2022). Meanwhile, the mitogen-activated protein kinase (MAPK) pathway induces the secretion of active matrix metalloproteinases (MMPs), resulting in vascular endothelial cell apoptosis and ultrastructural disruption (Lee et al., 2024). Inflammation also alters the expression and localization of TJs. TJs are composed of membrane proteins (e.g., occludin and claudin) and cytoplasmic accessory proteins (e.g., zonula occluden [ZO]-1 and ZO-2) (Feldman et al., 2005). MMPs, pro-inflammatory cytokines (e.g., IL-1β, IL-6, tumor necrosis factor-α [TNF-α], IL-12, and IL-23), chemokines (e.g., C-C motif chemokine ligand [CCL] 2, CCL4, and CCL3), complement components (e.g., Component [C] 1q and C3), and adhesion molecules (e.g., intercellular adhesion molecule-1 [ICAM-1], P-selectin, and E-selectin) activate brain microvascular endothelial cells (BMECs) through intrinsic signaling pathways and synergistically drive the migration of leukocytes, microglia, and perivascular macrophages into the perivascular space, contributing to remodeling of TJs (Andjelkovic et al., 2023). Among these, specific cytokines can affect the tight junction complex of brain microvascular endothelium through multiple pathways, leading to increased barrier permeability. For example, IL-6 activates signal transducer and activator of transcription 3 (STAT3) pathway, subsequently downregulating TJs at both the transcriptional and protein levels (occludin, ZO-1, claudin-5), accompanied by decreased transendothelial electrical resistance (TEER) and increased macromolecular permeability (Digiovanni et al., 2024). TNFα, for instance, inhibits claudin-5 promoter activity in brain endothelial cells (Aslam et al., 2012). TNFα results in a rapid and transient upward band-shift of occludin, downregulation of occludin expression, morphological changes in BMECs, and increased BBB permeability (Ni et al., 2017). Studies indicate that normal human brain microvascular endothelial cells exhibit an irregular stellate morphology. In contrast, TNFα-treated cells exhibit a spindle-shaped appearance, narrower and more elongated, with significantly reduced TEER measurements (Ni et al., 2017). The BBB serves as a critical gateway for peripheral-to-central inflammatory transmission. Its disruption—the breach of this gateway—allows inflammatory signals to enter the central nervous system.

Subsequently, the multicellular network within the NVU (including endothelial cells, microglia, astrocytes, pericytes, etc.) amplifies the central inflammatory response. Inflammation and complement activation induce microglia to transition into a pro-inflammatory state (M1 phenotype), releasing cytokines and chemokines that participate in multiple signaling pathways, including the C-C chemokine receptor type 5 (CCR5)-G-protein-coupled receptors (GPCRs)-Ras-MAPK pathway (Xu et al., 2023; Qi et al., 2024). Astrocytes also undergo morphological changes: their end-feet become swollen, connexin 43 in their gap junctions is damaged, and hemichannel activity increases, which can release harmful molecules into the extracellular space, thereby inducing neuroinflammation (Andjelkovic et al., 2023; Cho et al., 2021; Dong et al., 2022). Anesthesia and surgery induce MMP-9 activation, and MMP-9 degrades the extracellular matrix and cell adhesion molecules, detaching pericytes from the capillary wall, which leads to pericyte loss, increased transcytosis in brain endothelial cells, and exacerbated BBB leakage (Hu et al., 2024). Furthermore, interactions among microglia, astrocytes, and mast cells collectively amplify inflammation. For example, microglia release IL-1α, TNFα, and C1q to induce A1 neurotoxic reactive astrocytes, which in turn secrete neurotoxins that can induce the rapid death of neurons and oligodendrocytes. Additionally, there is potential crosstalk between astrocytes and microglia via C3/C3aR signaling, playing a key role in synapse elimination (Liddelow et al., 2017; Xiong et al., 2018). For instance, mast cells release histamine, tryptase, and various cytokines that can activate microglia. Microglia respond to mast cells through Toll-like receptor (TLR) 2/TLR4 signaling molecules, prompting mast cells to release cytokines that further activate microglia, thus forming a positive feedback loop (Skaper et al., 2014). As another example, mast cell proteases can induce the activation of astrocytes to release IL-33 via the p38/extracellular signal-regulated kinase (ERK)/NF-κB pathway. Astrocyte-derived IL-33, in turn, can prompt mast cells to release IL-6, IL-13, and CCL2 to modulate microglia (Huang et al., 2024; Skaper et al., 2014).

Perioperative BBB dysfunction and neuroinflammation impact proximal biological endpoints, including synapses and neural networks, through pathways such as the complement-microglia axis, receptor channels, and energy matching. Alterations in these endpoints are directly related to the cognitive phenotype of PNDs. For instance, the transformation of microglia to the M1 phenotype affects neuronal autophagy and mediates pathological synaptic pruning, leading to neuronal damage and cognitive decline (Qi et al., 2024; Xu et al., 2023). Furthermore, the disruption of interactions between neurons, astrocytes, and cerebral blood vessels (i.e., neurovascular coupling) impairs synaptic plasticity, affecting the release of neurotransmitters, neuromodulators, and vasoactive mediators, as well as normal brain function (Andjelkovic et al., 2023; Cho et al., 2021; Dong et al., 2022).

BBB dysfunction is a critical link in the influx and amplification of inflammatory signals during the pathogenesis of PNDs and should be a priority target for intervention. Current pharmacological strategies for PNDs focus on preserving BBB integrity and reducing neuroinflammation. Targeted agents include MMP inhibitors (e.g., GM6001), NF-κB antagonists, NLRP3/caspase-1 inhibitors, and mast cell stabilizers (e.g., cromoglycate) (Zhang et al., 2016; Cheng et al., 2022; Zhu et al., 2024). Additional anti-inflammatory agents, such as minocycline and non-steroidal anti-inflammatory drugs (e.g., ibuprofen), are also under investigation (Cheng et al., 2022). Non-pharmacological interventions, such as electroacupuncture preconditioning, may reduce oxidative stress and neuroinflammation by preserving the function of telomerase reverse transcriptase, potentially alleviating PNDs (Wang et al., 2024). While some animal studies suggest therapeutic efficacy, most approaches remain in preclinical stages. Further research is needed to confirm their clinical relevance in treating human PNDs.

## 3 Neurotransmitter or receptor abnormalities and the BBB

Deficits in the cholinergic system are key contributors to PNDs. Anesthesia and surgery reduce the number of cholinergic neurons and the expression of acetylcholine receptors like the α7 nicotinic acetylcholine receptor (α7-nAChR) in aged mice (Xu et al., 2019; Wei et al., 2022). α7-nAChR serves as a positive regulator of TJ expression in the BBB. As a positive regulator of TJ expression, selective activation of α7-nAChR increases claudin-5 and occludin levels, thereby strengthening the endothelial barrier (Kimura et al., 2019). Therefore, perioperative cholinergic deficits may directly increase BBB permeability.

Acetylcholine and its receptors also regulate the BBB via astrocytes. α7-nAChR activation in astrocytes reduces the secretion of inflammatory cytokines by inhibiting the NF-κB pathway (Patel et al., 2017). Strategies to enhance overall cholinergic signaling, such as using acetylcholinesterase inhibitors like tacrine, which reduce acetylcholine degradation in the synaptic cleft, have been shown to potentially play a significant role in the treatment of PNDs (Kotekar et al., 2018). In contrast, α7-nAChR inhibition has been reported to promote A2 astrocyte formation, enhance the release of neurotrophic factors, and increase glutamate uptake by inhibiting the NF-κB pathway (Chen et al., 2024). This apparent contradiction suggests that the function of α7-nAChR in PNDs may be highly dependent on the cell’s location, timing, and context (Letsinger et al., 2022). Consequently, future therapeutic strategies targeting this receptor require greater precision to modulate α7-nAChR function under specific temporal and signaling conditions.

Glutamate dysregulation also contributes to PNDs. Astrocytes normally maintain low extracellular glutamate levels, but their clearance capacity decreases perioperatively (Cao et al., 2022). Following peripheral surgery, reduced expression of glutamine synthetase (GS) and the glutamate-aspartate transporter (GLAST) in mouse hippocampal astrocytes impairs extracellular glutamate uptake and conversion (Femenía et al., 2018). Decreased expression of astrocyte glutamate transporter 1 (GLT-1) in hippocampal astrocytes further reduces glutamate clearance, increasing susceptibility to PNDs (Jiao et al., 2024).

Excess glutamate activates N-methyl-D-aspartate receptors (NMDARs), leading to endothelial damage and the suppression of claudin-5 and occludin via the Ras homolog (Rho)/Rho-associated kinase (ROCK) pathway, thus compromising BBB integrity (Yu et al., 2022). Elevated extracellular glutamate levels also activate astrocytes and promote the release of inflammatory factors, while deleting NMDAR subunit 2C in astrocytes alleviates neuroinflammation (Zuo et al., 2023; Gao et al., 2024). These findings suggest that NMDAR antagonists like amantadine may be potential therapeutic agents for PNDs (Alaçam et al., 2025).

## 4 Aβ deposition, tau protein phosphorylation and the BBB

Excessive Aβ deposition and tau protein phosphorylation initiate pathogenic cascades that damage surrounding neurons and synapses, contributing to cognitive dysfunction (Yang et al., 2022). In animal models of PNDs, increased central expression of Aβ and phosphorylated tau (p-tau) has been observed (Chen et al., 2022).

Aβ1-42, the most common Aβ isoform associated with PNDs, increases BMEC permeability and induces mitochondrial dysfunction, which is characterized by elevated reactive oxygen species (ROS) and reduced adenosine triphosphate (ATP) levels (Zhao et al., 2025). On the other hand, alterations in the structure and function of the BBB also affect the transport of Aβ. In aged PND mice induced by Sevoflurane, the receptor for advanced glycation end products (RAGE) is overexpressed in hippocampal endothelial cells (Liang et al., 2020). RAGE serves as the primary influx transporter for free Aβ from the circulation into the central nervous system. Peripheral HMGB1 crosses the compromised BBB and binds to TLR4 on endothelial cell membranes, thereby promoting RAGE transcription and translation, which leads to the excessive transport of plasma Aβ into the brain (Irzan et al., 2025; Li et al., 2025). BBB disruption also impairs multiple Aβ efflux transporters, such as low-density lipoprotein receptor-related protein 1 (LRP1) and P-glycoprotein (P-gp), through pathways including neuroinflammation (Versele et al., 2022; Huang et al., 2019). This ultimately forms a vicious cycle: Aβ damages the BBB, which in turn impairs Aβ transport, leading to further accumulation. In ApoE4 transgenic knock-in mice, cognitive impairment is more pronounced after surgery and is accompanied by greater Aβ accumulation in the hippocampal CA3 region (Kim et al., 2021). ApoE4 is a high-risk subtype of cerebral lipid transporters that not only affects cholesterol homeostasis but also specifically modulates Aβ-receptor interactions (Chen et al., 2025). ApoE4 binds to Aβ to form Aβ-ApoE4 complexes, which inhibit LRP1-mediated Aβ efflux transport in endothelial cells and further exacerbate Aβ deposition in the feedback loop (Zhou et al., 2023; Zhang et al., 2022). As a potential therapeutic, graphene oxide-based nanomaterials have been shown to inhibit the β-cleavage of amyloid precursor protein and improve the delivery of endosomal Aβ to lysosomes, thereby reducing Aβ generation and enhancing its degradation, which significantly lowers hippocampal Aβ levels and improves cognitive function in postoperative mice (Zhang et al., 2020).

Tau proteins can also disrupt BBB integrity independently of Aβ. Inhibiting tau expression with doxycycline restores BBB integrity (Blair et al., 2015). Varenicline mitigates tau protein mislocalization and improves cognitive outcomes (Wang J. et al., 2022).

However, the precise contributions of Aβ and tau to PNDs remain uncertain. Among community-dwelling older adults carrying the ApoE4 allele, preoperative functional connectivity in Alzheimer’s disease–vulnerable brain regions is elevated, followed by a steep postoperative decline. Nevertheless, cerebrospinal fluid levels of Aβ, tau, or p-tau, and cognitive trajectories, do not significantly differ between ApoE4 carriers and non-carriers (Browndyke et al., 2021). Furthermore, in cognitively intact older adults undergoing non-cardiac, non-neurological surgery, no consistent associations have been found between neurocognitive outcomes and postoperative Aβ or tau fluctuations (Berger et al., 2022). These findings indicate that Aβ and tau protein phosphorylation may contribute to the pathogenesis of PNDs in some populations. However, their role requires further investigation.

## 5 Oxidative stress and the BBB

Oxidative stress increases with age, surgery, and anesthesia, disrupting both the structure and function of the BBB through multiple pathways implicated in the development of PNDs. The detrimental effect of oxidative stress is first evident in the compromised integrity of TJs. Under hypoxia/reoxygenation conditions, ROS downregulate TJs, including claudin-5, occludin, and ZO-1, in BMECs, thereby increasing BBB permeability (Won et al., 2015). Free radical scavengers like secoisolariciresinol diglucoside (SDG) can attenuate hypoxia-induced BBB dysfunction and maintain tight junction integrity, highlighting the importance of ROS scavenging in preserving the barrier (Nzou et al., 2020).

Activated MMPs play a crucial role in degrading the proteins of the BBB basement membranes and tight junctions. Activated MMPs degrade TJs and the basement membranes, while ROS may regulate BBB permeability in aged mice via the cyclophilin A (CyPA)/MMP-9 pathway (Liu et al., 2022). ROS also induce cytoskeletal rearrangement via the ROS/Ras homolog family member A (RhoA)/phosphoinositide 3-kinase (PI3K)/protein kinase B (PKB) signaling cascade, further weakening barrier performance (Gu et al., 2013). Excessive ROS can also directly interfere with the key substance transport functions of BMECs. *In vitro* experiments have demonstrated that ROS accumulation within endothelial cells activates the intracellular ERK1/2 signaling pathway, significantly downregulating the mRNA expression of the ATP-binding cassette transporter ABCG2 and hindering its mediated efflux of neurotoxic substances, while antioxidants (e.g., N-acetylcysteine) or ERK1/2 inhibitors (e.g., U0126) can effectively reverse this downregulation (Newman et al., 2023). Excess ROS also compromise mitochondrial respiration and morphology in brain capillaries, impairing energy metabolism and survival of vascular endothelial cells (Velmurugan et al., 2024). Mitochondrial morphological homeostasis depends on the dynamic balance between fission and fusion, and oxidative stress is a key factor that drives excessive mitochondrial fission. Research has found that ROS can induce the translocation of coactivator-associated arginine methyltransferase 1 (CARM1) to the cytoplasm, where CARM1 methylates dynamin-related protein 1 (DRP1), triggering excessive mitochondrial fission. This, in turn, forms a positive feedback loop of ROS generation (i.e., the CARM1-DRP1-ROS axis), continuously reinforcing the mitochondrial morphological imbalance and thereby accelerating dysfunction and cellular senescence (Cho and Kim, 2024). Additionally, studies have found that mitochondria-targeted therapies (e.g., Mitoquinone) can effectively activate the AMP-activated protein kinase (AMPK) signaling pathway, upregulating the expression of key transcription factors Sirt1 and Pgc1α. This significantly improves mitochondrial respiratory function, thereby restoring cellular metabolic homeostasis and effectively reversing ROS-induced damage (Cai et al., 2025). Moreover, ROS can further activate NLRP3 through the binding of thioredoxin-interacting protein (TXNIP) to the NLRP3 inflammasome, inducing the production of IL-1β, which exacerbates degradation of TJs and barrier dysfunction (Cao et al., 2016).

In summary, oxidative stress is a key pathological basis for PNDs. ROS employ multi-target mechanisms that lead to both structural leakage and functional impairment of the BBB. Currently, antioxidant therapies have shown neuroprotective potential (Wang W. et al., 2023; Wang Y. et al., 2023), and future research should focus on two main directions. First, targeted mechanistic studies should be conducted to systematically analyze the key signaling axes of ROS-mediated BBB damage (e.g., the CARM1–DRP1–ROS pathway). Second, based on clearly identified pathological targets, emphasis should be placed on developing mitochondria-targeted antioxidants with BBB-penetrating capabilities (e.g., Mitoquinone) and combining anti-inflammatory and antioxidant interventions based on these mechanistic insights.

## 6 Iron dysregulation and the BBB

Iron is essential for normal brain function, and disruptions in its uptake, transport, storage, or utilization can lead to excessive intracellular accumulation of ferrous iron (Fe^2+^). Such excessive iron accumulation is common in neurological disorders such as Alzheimer’s disease, Parkinson’s disease, and other disorders that manifest neurodegeneration and associated brain iron accumulation (Reichert et al., 2020). In a laparotomy model of mice, brain iron overload and altered expression of iron metabolism-related proteins were observed for up to 14 days after surgery (Li et al., 2016). Another mouse study reported that sevoflurane induces apoptosis through cross-dysregulation of iron and glucose metabolism and induction of energy stress, implicating iron–glucose imbalance in sevoflurane-induced cognitive impairment (Ge et al., 2021). These metabolic changes occurred concurrently with cognitive decline. Increased iron deposition in the brain (especially the hippocampus) and oxidative stress are related to the pathogenesis of PNDs.

Iron dysregulation disrupts the BBB integrity by affecting multiple components of the NVU. Iron-induced ROS downregulate TJs and induce BMEC apoptosis. Mitochondrial ferritin (FtMt) protects against TJ loss and apoptosis by inhibiting iron dysregulation and ROS accumulation in BMECs (Wang P. et al., 2022). Vascular endothelial (VE)-cadherin is a key regulator of endothelial permeability. Additionally, an increase in the labile iron pool (LIP) reduces the expression of VE-cadherin on endothelial cell surfaces through hypoxia-inducible factor 2α (HIF2α), while the iron chelator Desferal (DFO) preserves BBB integrity via HIF2α regulation (Rand et al., 2021). Astrocytes respond to elevated intracellular iron by secreting hepcidin, which reduces iron influx by decreasing ferroportin 1 (FPN1) expression on endothelial cells, a mechanism that may mitigate BBB dysfunction in neurodegenerative diseases (You et al., 2022). DFO significantly reduces hippocampal iron concentration and ferritin levels in rats with PNDs, and reverses surgery-induced abnormal changes in iron transport-related proteins, suggesting that iron chelators may be a candidate strategy for the intervention of PNDs (Pan et al., 2016).

## 7 Disrupted circadian rhythm and the BBB

Circadian rhythm disruption refers to a misalignment between an organism’s internal biological clock and external environmental cues. Aging, a major risk factor for PNDs, is associated with dysregulation of core circadian genes, including the brain and muscle ARNT-like protein 1 (BMAL1) gene and the circadian locomotor output cycle kaput (CLOCK) gene (Wyse and Coogan, 2010). Disruption of circadian rhythms activates astrocytes and impairs pericyte and endothelial cell function, a systemic cellular dysregulation directly destabilizing the BBB. The role of abnormal clock gene expression in astrocyte activation and central nervous system degeneration remains controversial. For example, specific BMAL1 deletion can activate astrocytes and increase the expression of the autophagy chaperone Bcl2-associated athanogene 3 (Bag3), enhancing the phagocytosis of alpha-synuclein (αSyn) and tau proteins (Sheehan et al., 2023). Conversely, aberrant clock gene expression may exacerbate astrocyte activation and neuroinflammation, while BMAL1 activation can attenuate inflammation and alleviate PND symptoms (Hou et al., 2019; Niu et al., 2022). Thus, clock gene dysregulation appears to affect the inflammatory/phagocytic balance in astrocytes. Circadian disruption also directly impairs BBB structural integrity. Altered expression of BMAL1 gene and CLOCK gene can reduce the expression of claudin-5 and occludin at both the mRNA and protein levels (Tiburcio et al., 2024). BMAL1 deficiency also induces pericyte dysfunction by decreasing platelet-derived growth factor receptor β (PDGFRβ), a key regulator of BBB integrity, thereby contributing to age-dependent BBB breakdown, reduced cerebral blood flow, and increased accumulation of blood-derived neurotoxins (Nakazato et al., 2017). Therefore, the disruption of endothelial tight junctions and impaired pericyte function, driven by the dysregulation of clock genes, collectively lead to the breakdown and dysfunction of the physical BBB. Notably, circadian rhythms may also affect the BBB indirectly via the gut–brain axis. Disruption of gut microbiota homeostasis impairs gut barrier integrity and promotes BBB dysfunction (Li et al., 2021).

Preoperative melatonin administration mitigates the impact of surgery and anesthesia on the expression of melatonin receptor and clock gene, such as BMAL1, which improves postoperative neurobehavioral outcomes in aged mice (Jia et al., 2024). Additionally, nobiletin, an enhancer of the clock protein retinoic acid receptor-related orphan receptors (RORs), may protect against PNDs by supporting the expression of BMAL1 and RORs (Sun et al., 2022). A deeper understanding of circadian regulation of the BBB may therefore inform novel therapeutic strategies for PNDs.

## 8 The microbial–gut–brain axis and the BBB

The gut microbiota is implicated in neurodegenerative disorders through the microbiota–gut–brain (MGB) axis. Surgery and anesthesia can induce gut dysbiosis, contributing to PNDs primarily through two mechanisms: reduced production of beneficial metabolites and increased production of harmful compounds.

First, dysbiosis of the intestinal flora reduces beneficial metabolites of short-chain fatty acids (SCFAs), such as acetic acid, propionic acid, and butyrate. Reduced butyrate levels downregulate TJs and increase BBB permeability, contributing to PNDs. Butyrate supplementation has been shown to improve postoperative cognitive function (Wen et al., 2020). Second, dysbiosis may elevate harmful metabolites, including lipopolysaccharides, palmitamide, and trimethylamine N-oxide (TMAO), which damage the BBB and promote cognitive decline (Pan et al., 2023; Xiong et al., 2024). For example, TMAO is produced in the liver via the oxidation of trimethylamine, which is produced by the gut microbiota through the breakdown of dietary choline and carnitine. Gut TMAO levels are positively correlated with the severity of cognitive decline; TMAO is highly susceptible to crossing the BBB to reach the brain and may be involved in the development of PNDs by modulating microglial and astrocyte activation (Brunt et al., 2021; Xiong et al., 2024). Additionally, gut dysbiosis can activate immune pathways that influence PNDs. For example, it activates gut-derived T helper 17 (Th17) cells and increases IL-17 secretion. IL-17A upregulates its receptors on brain endothelial cells, elevates MMP levels, and reduces TJ expression, compromising BBB integrity and contributing to cognitive dysfunction (Ni et al., 2018; Wen et al., 2022). In summary, the microbiota-gut-brain axis, through its metabolic and immune-mediated pathways, acts on the BBB and disrupts its integrity. This positions the BBB as a key hub connecting perioperative dysbiosis to the central neuroinflammation that drives PNDs.

Preclinical studies have shown that cognitive function in patients with PNDs can be improved by gut microbiota modulation (e.g., probiotics), supplementation with beneficial metabolites (e.g., propionic acid pretreatment), dietary restriction, and intestinal barrier protection. These findings suggest that targeting the MGB axis could be a promising therapeutic strategy for PNDs. Identifying specific gut flora biomarkers may aid in screening at-risk populations and guiding interventions. However, current studies are limited and require further clinical validation and standardized implementation (Sugita et al., 2023; Dai et al., 2024; Ren et al., 2024).

## 9 Discussion

The pathogenesis of PNDs does not stem from a single pathological mechanism but rather a complex regulatory network centered on the dysfunction of the BBB. The various mechanisms systematically described in this paper demonstrate significant crosstalk and synergistic amplification effects. First, systemic inflammation induced by perioperative stress is a key initiating factor. By activating signaling pathways such as NF-κB and MAPK, it damages the tight junctions between cerebral microvascular endothelial cells, leading to increased BBB permeability (Cheng et al., 2022; Lee et al., 2024). The infiltration of peripheral inflammatory mediators activates microglia and astrocytes, creating a vicious cycle in which inflammation compromises BBB integrity, which in turn exacerbates the inflammatory response. Building on this core feedback loop, other pathological processes also contribute synergistically to BBB damage through mutual crosstalk. For instance, Aβ metabolic imbalance has a mutually reinforcing relationship with neuroinflammation. On the one hand, abnormal Aβ deposition can activate glial cells, leading to inflammatory responses (Hansen et al., 2018). On the other hand, the inflammatory microenvironment impedes Aβ efflux by downregulating Aβ clearance transporters such as P-gp, leading to endothelial cell damage and BBB leakage (Zhang et al., 2022; Zhao et al., 2025). Meanwhile, glutamate excitotoxicity and cholinergic deficits affect barrier integrity by altering the endothelial cytoskeleton and inflammatory factor secretion through the NMDAR-Rho/ROCK and α7-nAChR pathways, respectively (Yu et al., 2022; Patel et al., 2017). Iron-mediated ROS can induce the downregulation of TJs and the apoptosis of BMECs; furthermore, an increase in LIP can lead to a reduction in HIF2α-mediated VE-cadherin expression on the endothelial cell surface, collectively leading to BBB dysfunction (Wang P. et al., 2022; Rand et al., 2021). ROS can regulate BBB permeability through multiple inflammatory factor pathways. For example, ROS can further activate NLRP3 by binding TXNIP to the NLRP3 inflammasome, inducing the production of IL-1β which promotes degradation of TJs (Cao et al., 2016). Dysregulated expression of the core clock gene BMAL1 due to circadian rhythm disruption reduces the activity of efflux transporters, lowering the clearance efficiency of neurotoxic substances like Aβ, while also exacerbating astrocyte activation and increasing central inflammation levels, amplifying the damage to the BBB from upstream inflammatory signals (Niu et al., 2022; Hou et al., 2019; Zhang et al., 2021). Regarding the gut-brain axis, Th17-IL-17A signaling downregulates TJs (Ni et al., 2018; Wen et al., 2022), and gut dysbiosis leads to increased TMAO production which promotes the activation of microglia and astrocytes (Xiong et al., 2024; Brunt et al., 2021), all of which directly or indirectly lead to BBB damage. In summary, the pathophysiological network of PNDs is characterized by multiple mechanisms converging on BBB damage through various pathways. Whether it is systemic inflammation, oxidative stress, central protein or ion metabolism disorders, distal gut dysbiosis, or circadian rhythm dysregulation, their pathogenic signals ultimately all point to the disruption of BBB integrity. This highlights the pivotal role of the BBB as a key hub linking multi-dimensional pathophysiological changes with the final cognitive outcome.

The structural and functional integrity of the BBB is essential for maintaining brain homeostasis. Multiple perioperative factors can impair BBB function, contributing to the onset and progression of PNDs. Numerous studies have explored therapeutic strategies aimed at preserving BBB function to prevent or treat PNDs. For example, the perioperative use of melatonin, which has been shown to reduce the incidence of PNDs in elderly patients (Fan et al., 2017); early monitoring of iron homeostasis and iron chelator, which may offer a novel treatment option for PNDs (Li et al., 2016; Pan et al., 2016); and the application of inflammation-related enzyme inhibitors and cytokine antagonists, are effective in animal models (Cheng et al., 2022; Zhu et al., 2024). Acetylcholinesterase inhibitors and NMDAR antagonists are also considered potential treatment options for PNDs due to their effects on neurotransmitter pathways (Kotekar et al., 2018; Alaçam et al., 2025). Moreover, strategies targeting Aβ deposition and tau protein phosphorylation represent another promising avenue (Zhang et al., 2020; Wang J. et al., 2022). Additionally, antioxidant therapies and gut microbiota remodeling have likewise shown neuroprotective effects in preclinical models (Wang W. et al., 2023; Wang Y. et al., 2023; Sugita et al., 2023; Dai et al., 2024; Ren et al., 2024).

This review also has certain limitations. First, it is a narrative review, not a systematic one, so the literature selection may be subject to a certain bias and may not have covered all relevant research. Second, most of these approaches are supported only by animal studies, and their efficacy and safety in humans remain uncertain. For example, differences between human and animal gut microbiota limit the generalizability of findings on gut microbiota remodeling. Finally, while this review elaborates on the central role of the BBB in the multiple mechanisms of PNDs, it does not explore with the same depth the precise initial signaling pathways through which perioperative stimuli, such as surgery and anesthesia, initiate the first step of BBB disruption. These limitations indicate the need for more systematic reviews in the future.

Although the role of BBB dysfunction in PNDs is evident, few studies have directly investigated how BBB damage mediates the development of PNDs, a topic worth further research. While the mechanisms underlying PNDs, particularly neuroinflammation, are increasingly understood, large-scale clinical evidence evaluating relevant interventions is lacking. Key areas such as drug selection, dosing regimens, and perioperative implementation strategies remain underexplored. Clarifying these aspects is essential for bridging the gap between experimental findings and clinical application. Despite these gaps, the current evidence suggests that multiple mechanistic pathways hold promise for understanding and preventing PNDs. Continued research will be instrumental in advancing our mechanistic understanding and the development of effective clinical interventions.
